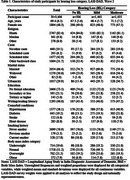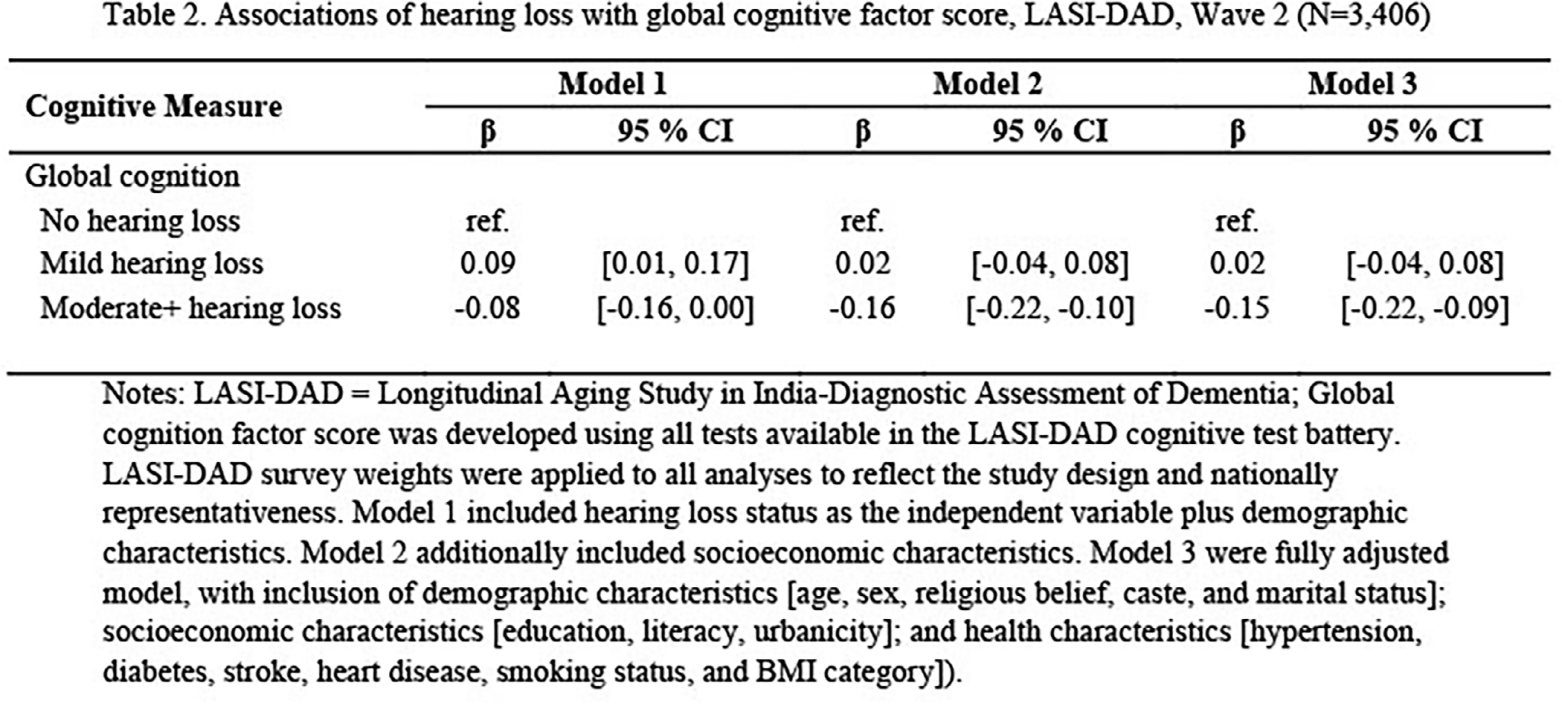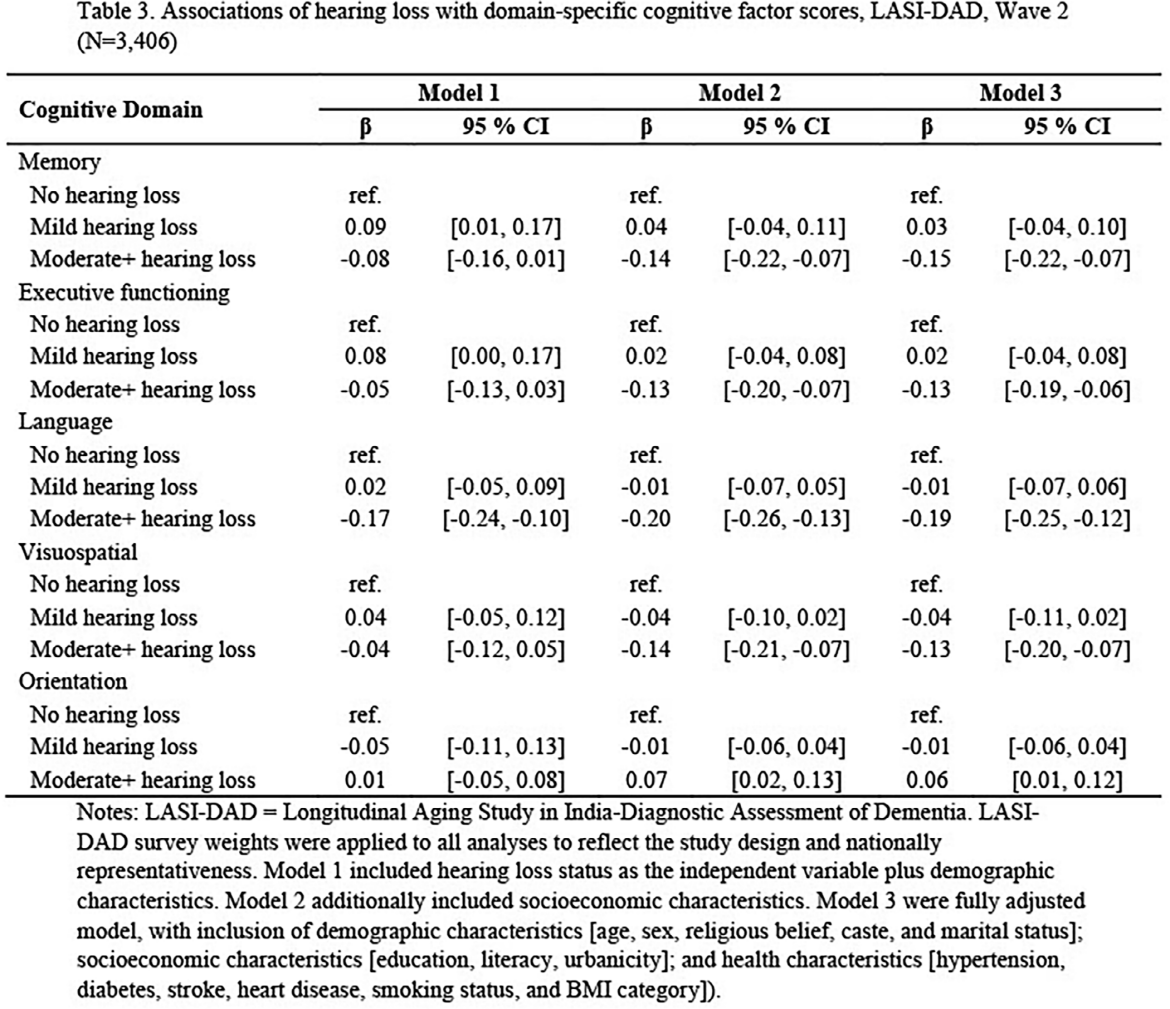# Hearing Loss and Cognition Among Older Adults in the Longitudinal Aging Study in India‐Diagnostic Assessment of Dementia (LASI‐DAD) Study

**DOI:** 10.1002/alz70860_099413

**Published:** 2025-12-23

**Authors:** Nicholas S Reed, Wuyang Zhang, David Li, Emma Nichols, A.B Dey, Jinkook Lee

**Affiliations:** ^1^ New York University, New York, NY, USA; ^2^ Johns Hopkins University, Baltimore, MD, USA; ^3^ University of Southern California, Los Angeles, CA, USA; ^4^ Department of Geriatric medicine, All India Institute of Medical Sciences, New Delhi, National Capital Territory of Delhi (NCT), India; ^5^ Center for Economic and Social Research, University of Southern California, Los Angeles, CA, USA

## Abstract

**Background:**

Numerous recent epidemiologic studies suggest hearing loss is associated with cognitive decline among older adults. However, these studies are mainly limited to the United States and Western Europe and often rely on self‐report hearing which is subject to bias related to the outcome of interest (e.g., cognition).

**Method:**

The Harmonized Diagnostic Assessment of Dementia for the Longitudinal Aging Study in India (LASI‐DAD), a nationally representative study of Indiana ≥ 60 years, added pure‐tone audiometry (portable HearX audiometer; trained field technicians) in wave 2 (2022‐2024). Hearing loss was measured continuously and categorically per World Health Organization Criteria (four‐frequency pure‐tone average [500,1000,2000,4000Hz] greater than 25dB). Cognitive function was assessed using overall (global) and domain‐specific factor scores (memory, executive functioning, language, visuospatial) derived from a cognitive test battery (Gross et al., 2020). Descriptive statistics and multivariate linear regression were used to characterize the association between hearing and cognition. Models included age, sex, caste, marital status, education, urbanicity, literacy level, and health‐related factors [BMI, smoking status, and self‐reported diagnoses of diabetes, stroke, hypertension, and heart disease].

**Result:**

Among 3406 participants with a hearing assessment (mean age=69.4 years; SD=6.5), 506 had no hearing loss (67.3 years; 5.6), 1465 had mild hearing loss (68.4 years, 5.7), and 1435 had moderate hearing loss (71.2 years; 7.2). In fully adjusted linear regression models, moderate (β=‐0.15;95%Confidence Interval[CI]=‐0.22:‐0.07) but not mild (β=0.03;95%CI=‐0.04:0.10) hearing loss was associated with reduced global cognitive scores relative to those without hearing loss, respectively. Similarly, an association between lower cognitive scores and moderate, but not mild, hearing loss were found the memory (β=‐0.15;95%CI=‐0.22:‐0.07), executive function (β=‐0.13;95%CI=–0.19:‐0.06), visuospatial (β=‐0.13;95%CI=‐0.20:‐0.07), and language (β=‐0.19;95%CI=‐0.25:‐0.12) domains.

**Conclusion:**

In a first‐in‐kind nationally representative sample of older Indians, moderate or greater hearing loss was associated with poorer cognitive scores. Further investigation in the LASI‐DAD sample and replication studies are needed to better understand how hearing impacts healthy aging in India and other countries across the globe.

Reference: Gross et al. Measurement and Structure of Cognition in the Longitudinal Aging Study in India‐Diagnostic Assessment of Dementia. J Am Geriatr Soc. 2020 Aug;68 Suppl 3(Suppl 3):S11‐S19. doi: 10.1111/jgs.16738.